# Cardiac surgery for patients with heart failure due to structural heart disease in Uganda: access to surgery and outcomes

**DOI:** 10.5830/CVJA-2014-034

**Published:** 2014

**Authors:** Antonio Grimaldi, Enrico Ammirati, Anna Chiara Vermi, Annalisa De Concilio, Giorgio Trucco, Francesco Aloi, Francesco Arioli, Filippo Figini, Santo Ferrarello, Francesco Maria Sacco, Renato Grottola, Paul G D’Arbela, Eloi Marijon, Mariana Mirabel, Ottavio Alfieri, Nicole Karam, Juergen Freers

**Affiliations:** St Raphael of St Francis, Nsambya Hospital, Kampala, Uganda; Cardiovascular and Thoracic Department, San Raffaele Hospital, Milan, Italy; St Raphael of St Francis, Nsambya Hospital, Kampala, Uganda; Cardiovascular and Thoracic Department, San Raffaele Hospital, Milan, Italy; St Raphael of St Francis, Nsambya Hospital, Kampala, Uganda; Cardiovascular and Thoracic Department, San Raffaele Hospital, Milan, Italy; St Raphael of St Francis, Nsambya Hospital, Kampala, Uganda; St Raphael of St Francis, Nsambya Hospital, Kampala, Uganda; St Raphael of St Francis, Nsambya Hospital, Kampala, Uganda; St Raphael of St Francis, Nsambya Hospital, Kampala, Uganda; Cardiovascular and Thoracic Department, San Raffaele Hospital, Milan, Italy; St Raphael of St Francis, Nsambya Hospital, Kampala, Uganda; Cardiovascular and Thoracic Department, San Raffaele Hospital, Milan, Italy; St Raphael of St Francis, Nsambya Hospital, Kampala, Uganda; Cardiovascular and Thoracic Department, San Raffaele Hospital, Milan, Italy; St Raphael of St Francis, Nsambya Hospital, Kampala, Uganda; Cardiovascular and Thoracic Department, San Raffaele Hospital, Milan, Italy; St Raphael of St Francis, Nsambya Hospital, Kampala, Uganda; St Raphael of St Francis, Nsambya Hospital, Kampala, Uganda; St Raphael of St Francis, Nsambya Hospital, Kampala, Uganda; Paris Cardiovascular Research Centre, INSERM U970, Paris, France; St Raphael of St Francis, Nsambya Hospital, Kampala, Uganda; Paris Cardiovascular Research Centre, INSERM U970, Paris, France; Cardiovascular and Thoracic Department, San Raffaele Hospital, Milan, Italy; Paris Cardiovascular Research Centre, INSERM U970, Paris, France; Division of Cardiology, Department of Medicine, Makerere University, Kampala, Uganda

**Keywords:** heart failure, rheumatic heart disease, congenital heart disease, echocardiography, heart surgery

## Abstract

**Objective:**

Few data are available on heart failure (HF) in sub-Saharan Africa. We aimed to provide a current picture of HF aetiologies in urban Uganda, access to heart surgery, and outcomes.

**Methods:**

We prospectively collected clinical and echocardiographic data from 272 consecutive patients referred for suspected heart disease to a tertiary hospital in Kampala during seven non-governmental organisation (NGO) missions from 2009 to 2013. We focused the analysis on 140 patients who fulfilled standardised criteria of HF by echocardiography.

**Results:**

Rheumatic heart disease (RHD) was the leading cause of HF in 44 (31%) patients. Among the 50 children included (age ≤ 16 years), congenital heart disease (CHD) was the first cause of HF (30 patients, 60%), followed by RHD (16 patients, 32%). RHD was the main cause of HF (30%) among the 90 adults. All 85 patients with RHD and CHD presented with an indication for heart surgery, of which 74 patients were deemed fit for intervention. Surgery was scheduled in 38 patients with RHD [86%, median age 19 years (IQR: 12–31)] and in 36 patients with CHD [88%, median age 4 years (IQR 1–5)]. Twenty-seven candidates (32%) were operated on after a median waiting time of 10 months (IQR 6–21). Sixteen (19%) had died after a median of 38 months (IQR 5–52); 19 (22%) were lost to follow up.

**Conclusions:**

RHD still represents the leading cause of HF in Uganda, in spite of cost-efficient prevention strategies. The majority of surgical candidates, albeit young, do not have access to treatment and present high mortality rates.

## Abstract

Improvement in the control of infectious diseases and malnutrition associated with changes in lifestyle has led to a new epidemiological pattern in many low- and middle-income countries. Non-communicable diseases, mainly cardiovascular disorders, have emerged as major causes of morbidity and mortality in most sub-Saharan African countries.^1^ Hospital-based studies indicate that heart failure (HF) accounts for 3–7% of all admissions to African hospitals.^2^,^3^-^7^

Although there has been increasing interest in the epidemiology of cardiovascular diseases in the African continent,^7^-^9^ recent data from Uganda are scarce^7^,^10^ but most needed to guide public health policies. Most registers originate from South Africa and cannot be transposed to poorer sub-Saharan countries.^2^,^11^

Echocardiography is a mainstay in the assessment of HF. Unfortunately, access to echocardiography remains limited in many African countries due to cost and lack of skilled health workers, thereby leading to little data on cardiovascular diseases.^12^

We report on the distinctive patterns of HF through a prospective, cross-sectional, hospital-based study in patients referred for suspected heart disease in urban Kampala, Uganda, in order to characterise the features of HF and to tailor future interventions. We also aimed at assessing access to invasive interventions and outcomes in patients with surgical indications.

## Methods

## Study setting

The study was conducted at the St Raphael of St Francis Nsambya Hospital, a tertiary, non-profit hospital with a capacity of 361 beds, located in urban Kampala. Uganda has a population of 33 425 000 and a life expectancy of 48 and 57 years in males and females, respectively (http://www.who.int/countries/uga/en/)]. The Italian association Solidarity Among People (AISPO), a non-governmental organisation (NGO) managed by the San Raffaele Scientific Institute in Milan, Italy, conducted the project in co-operation with local medical staff.

The main objectives of the project were to gather epidemiological data on HF in Uganda, and to train Ugandan doctors, with a special focus on echocardiographic skills. The present study was conducted during seven NGO missions (cumulative period of 36 weeks from 2009 to 2013). The seventh mission was performed in 2013 in order to follow up on patients who had undergone surgery and those still on the waiting list. Patients were systematically evaluated by clinical and echocardiographic examination.

## Study cohort

We prospectively studied 272 consecutive subjects [median age 35 years, interquartile range (IQR) 17–58; 59% female] referred to the St Raphael of St Francis Nsambya Hospital for suspected heart disease. Patients were evaluated by clinical and echocardiographic examination. Electrocardiogram, chest X-ray, chest computerised tomography (CT) scan and venous Doppler examination of the inferior limbs were performed as needed.

**Fig. 1.**
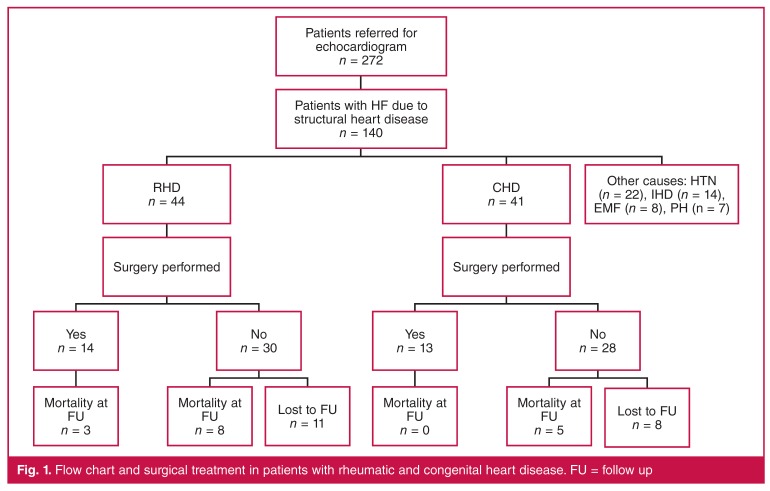
Flow chart and surgical treatment in patients with rheumatic and congenital heart disease. FU = follow up

We studied 160 out-patients (59%) and 112 in-patients (41%) from the general medical and paediatric wards. In the study population, 149 patients (55%) were female and 75 (27%) were children (≤ 16 years). Shortness of breath was the most frequent motive for seeking medical assistance (*n* = 114, 42%). One hundred and ninety-seven patients (72%) presented with structural heart disease, among which 140 (71%) were in clinical HF.^13^ The latter constituted the study cohort (Fig. 1).

## Echocardiographic evaluation and study definitions

Italian cardiology teams from the San Raffaele Scientific Institute in Milan carried out the echocardiograms. General Electric® Logic P5 machines with colour Doppler and two available probes (1.5–3.5 MHz for adults and 3–8 MHz for children) were used. Two experienced cardiologists reviewed all echocardiograms for definite diagnosis (AG and EA).

The aetiology of HF was assessed according to the European Society of Cardiology guidelines.^13^ HF was defined as systolic HF when left ventricular ejection fraction (LVEF) was < 50%; preserved ejection fraction HF when signs of increased left ventricular filling were detected; and right ventricular HF when the right ventricle was primarily affected or dysfunctional due to pulmonary hypertension (PH) not associated with left-sided heart abnormalities.

Ischaemic heart disease (IHD) was suspected when clear wall motion abnormalities were observed (there was no cardiac catheterisation laboratory in Uganda at the time of the study). HF was defined as hypertensive when long-lasting history of systemic hypertension and typical echocardiographic features such as left ventricular concentric hypertrophy or impaired left ventricular inflow patterns were found. We identified patients as potential candidates for cardiac surgery according to the current guidelines.^14^

This study was approved by the St Raphael of St Francis Nsambya Hospital Ethical Committee (May 2009). It conformed to the Declaration of Helsinki and Good Clinical Practice.

## Statistical analysis

We performed descriptive statistics for the more frequently observed heart diseases. The results are reported as median and interquartile range, or as numbers and percentages, as appropriate.

## Results

## Causes of HF in the whole cohort and according to age

**Fig. 2.**
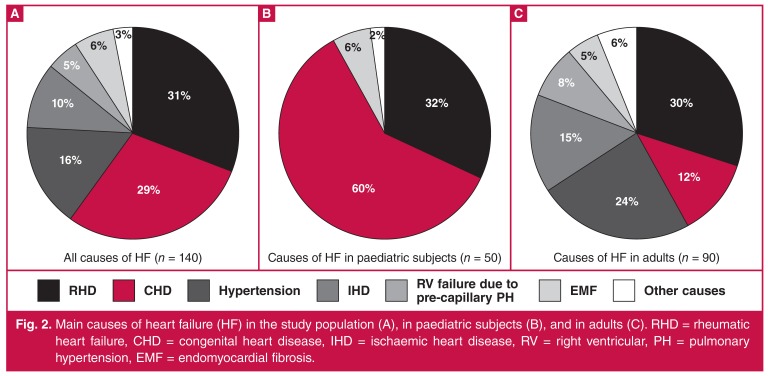
Main causes of heart failure (HF) in the study population (A), in paediatric subjects (B), and in adults (C). RHD = rheumatic heart failure, CHD = congenital heart disease, IHD = ischaemic heart disease, RV = right ventricular, PH = pulmonary hypertension, EMF = endomyocardial fibrosis.

The study group constituted a cohort of 140 patients with clinical HF. Median age was 40 years (IQR 14–66) and 83 (59%) were female. All patients were black Africans. The predominant cause of HF was RHD (*n* = 44; 31%) (Fig. 2A), mainly related to severe mitral regurgitation, which was either isolated or associated with multiple valve involvement. Other main causes of HF were CHD (*n* = 41; 29%), hypertensive cardiomyopathy (*n* = 22; 16%), highly suspected IHD (*n* = 14; 10%), endomyocardial fibrosis (EMF) (*n* = 8; 6%) and right ventricular failure due to pre-capillary PH (*n* = 7; 5%).

LVEF was reduced in 56 cases (40%). Moderate to severe right ventricular dysfunction was found in 70 (50%) cases. Clinical and echocardiographic characteristics of patients with HF are depicted in Table 1.

**Table 1:** Clinical and echocardiographic characteristic of 113 patients with heart failure.

Main cause of HF	*RHD*	*CHD*	*Hypertensive CMP*	*IHD*	*EMF*	*RVD associated with PH*	*Miscellaneous*	*Total*
No of cases (%)	44 (31)	41 (29)	22 (16)	14 (10)	8 (6)	7(5)	4 (3)	140
Age (years) [median (IQR)]	19 (12–52)	4 (1–17)	66 (56–76)	75 (60–85)	18 (14–30)	50 (38–72)	50 (32–66)	40 (14–66)
Females, *n* (%)	36 (82)	21 (58)	13 (59)	5 (36)	3 (37)	3 (43)	2 (50)	83 (59)
LV systolic dysfunction, *n* (%)	13 (29)	7 (17)	16 (73)	13 (93)	2 (25)	2 (28)	4 (100)	57 (41)
LVEF % [median (IQR)]	60 (40–60)	60 (57–60)	37 (20–55)	35 (25–40)	55 (45–60)	55 (48–58)	40 (30–40)	55 (35–60)
LV dilatation*, *n* (%)	30 (68)	10 (24)	12 (54)	8 (57)	1 (12)	0 (0)	3 (75)	64 (46)
LA severe dilatation^§^, *n* (%)	38 (86)	5 (12)	10 (45)	1 (7)	2 (25)	0 (0)	1 (25)	57 (41)
AF, *n* (%)	5 (11)	0 (0)	8 (36)	1 (7)	0 (0)	0 (0)	0 (0)	14 (10)
Moderate to severe MR, *n* (%)	39 (89)	10 (24)	12 (54)	5 (36)	6 (75)	2 (28)	2 (50)	76 (54)
PH, *n* (%)	43 (98)	34 (81)	20 (91)	7 (50)	7 (87)	7 (100)	2 (50)	120 (86)
Moderate to severe RV dysfunction, *n* (%)	27 (61)	20 (49)	12 (54)	3 (21)	8 (100)	6 (86)	1 (25)	77 (55)

RHD = rheumatic heart disease, CHD = congenital heart disease, CMP = cardiomyopathy, IHD = ischaemic heart disease, RVD = right ventricular dysfunction, PH = pulmonary hypertension, EMF = endomyocardial fibrosis, LV = left ventricle, EF = ejection fraction, LA = left atrium, AF = atrial fibrillation, MR= mitral regurgitation, RV= right ventricle. *Defined as end-diastolic diameter > 55 mm for adults. ^§^Defined as volume > 40 ml for adults, PH defined as pulmonary artery systolic pressure > 35 mmHg.

We further analysed causes of HF separately in children and adults (Fig. 2B, C). In the paediatric population [*n* = 50, age ≤ 16 years, median 6 (IQR 2–12)] CHD was the main cause of HF (*n* = 30; 60%), followed by RHD (*n* = 16; 32%). We also reported three cases of EMF (6%). In the first decade of life, CHD was the main cause of HF (29/31, 97%), while RHD was the most prevalent in the age group of 10 to 16 years (15/17, 94%).

In adults [*n* = 90, age > 16 years; median 55 (IQR 33–70)], RHD was the primary cause of HF (*n* = 27; 30%). Hypertensive cardiomyopathy and presumptive IHD were the most frequent causes of HF beyond the sixth decade of life. Overall, hypertensive cardiomyopathy and IHD ranged as second and third causes of HF in adults, 24 and 15%, respectively. Other causes are depicted in Fig. 2C.

## Rheumatic heart disease

RHD (*n* = 44) was the main cause of HF in adults and the second in children. Table 1 shows the echocardiographic features of RHD. The median age of patients with RHD complicated by HF was 19 years (12–52) with a female:male ratio of 2.1:1.

Briefly, the mitral valve was affected in all cases. Mitral regurgitation was the most common lesion (43/44 cases, 98%) and the degree of mitral regurgitation was often severe (29/43, 67%). Mitral stenosis was severe in 12 patients (27%). PH (i.e. pulmonary artery systolic pressures > 35 mmHg) was present in 43 subjects [98%; median 65 (50–70 mmHg)]. Moderate and severe right ventricular dysfunction was present in 27 patients (61%). Moderate to severe tricuspid regurgitation was present in 36 patients (82%) due to annular dilatation secondary to RV remodelling without significant rheumatic involvement.

**Fig. 3.**
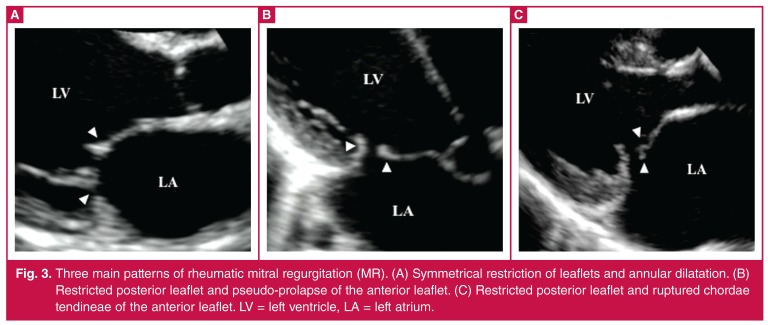
Three main patterns of rheumatic mitral regurgitation (MR). (A) Symmetrical restriction of leaflets and annular dilatation. (B) Restricted posterior leaflet and pseudo-prolapse of the anterior leaflet. (C) Restricted posterior leaflet and ruptured chordae tendineae of the anterior leaflet. LV = left ventricle, LA = left atrium.

Representative images of mitral lesions implicated in HF are shown in Fig. 3. We observed three main patterns of rheumatic mitral regurgitation: (1) symmetrical restriction of leaflets (30 cases, Fig. 3A); (2) posterior leaflet restriction and anterior leaflet pseudo-prolapse (eight cases, Fig. 3B); and (3) leaflet restriction and chordal rupture (five cases; Fig. 3C). Mitral lesions that did not appear calcified that were deemed suitable for surgical repair.

## Congenital heart disease

CHD (*n* = 41) was the main cause of HF in children under 16 years (30/50; 60%). Table 2 summarises the type of congenital defects observed. The main diseases associated with HF in children were isolated ventricular septal defect (VSD) (*n* = 7/30, 23%), atrio-ventricular septal defects (AVSD) (*n* = 4/30, 13%) and tetralogy of Fallot (*n* = 4/30, 13%). Other abnormalities included isolated atrial septal defects (ASD) (n = 6/41, 15%) and persistent ductus arteriosus (PDA) (*n* = 5/41, 12%).

**Table 2:** Types of congenital heart defects causing heart failure in the paediatric and adult populations.

*Type of defect, n (%)*	*≤ 16-year-old n = 30 (73)*	*>16-year-old n = 11 (27)*	*Total n = 41 (29)*
Simple defects	19 (63)	8 (73)	27 (66)
Atrial septal defect	3 (10)	3 (27)	6 (15)
Ventricular septal defect (VSD)	7 (23)	0 (0)	7 (17)
Atrio-ventricular septal defect	4 (13)	0 (0)	4 (10)
Congenital mitral cleft	1 (3)	0 (0)	1 (2)
Persistent ductus arteriosus	3 (10)	2 (18)	5 (12)
Congenital aortic regurgitation	0 (0)	1 (9)	1 (2)
RV outflow tract obstruction	1 (3)	2 (18)	3 (7)
Complex defects	11 (37)	3 (27)	14 (34)
Tetralogy of Fallot	4 (13)	1 (9)	5 (12)
VSD + pulmonary stenosis	1 (3)	0	1 (2)
VSD + tricuspid dysplasia	1 (3)	0	1 (2)
Univentricular heart	2 (7)	0	2 (5)
Persistent truncus arteriosus	2 (7)	0	2 (5)
Aorto-pulmonary window	1 (3)	0	1 (2)
Ebstein anomaly	0	2 (18)	2 (5)
Eisenmenger syndrome	0	2 (18)	2 (5)

Complex congenital defects were observed in 11 children (11/30, 37%), including rare diseases such as type II persistent truncus arteriosus and aortopulmonary window. Uncorrected CHD was responsible for HF in 11 adults (11/90; 12%), including two cases (5%) of Eisenmenger syndrome and two cases (5%) of severe Ebstein anomaly.

## Other aetiologies of structural heart disease

**Fig. 4.**
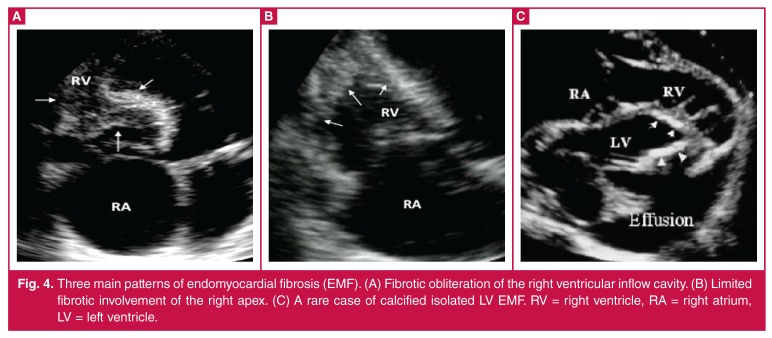
Three main patterns of endomyocardial fibrosis (EMF). (A) Fibrotic obliteration of the right ventricular inflow cavity. (B) Limited fibrotic involvement of the right apex. (C) A rare case of calcified isolated LV EMF. RV = right ventricle, RA = right atrium, LV = left ventricle.

We reported eight cases of EMF in HF (6% of all causes of HF in our series). The echocardiographic findings of biventricular EMF were similar in all cases, with the exception of the degree of right ventricular obstruction. In two cases, there was a significant obliteration of the right inflow tract and the apex (Fig. 4A). In the other case, the fibrosis involved only the right apex (Fig. 4B). We also observed a rare case of calcified isolated LV EMF (Fig. 4C).

The proportion of patients with HF associated with hypertension (*n* = 22/140; 16%) or presumptive IHD (*n* = 14/140; 10%) increased in older patients, with a peak incidence (33/52 among the 90 adults) in the seventh decade (Table 1). Pulmonary hypertension and right HF not associated with left-sided heart abnormalities accounted for 5% (7/140 cases) of causes of HF. Pulmonary embolism (6/7 cases, 86%) was recognised as the main cause of PH associated with right-sided HF. In four cases, CT scans or venous Doppler examination of the inferior limbs also supported the diagnosis.

## Patients with an indication for cardiac surgery: access to treatment, and outcomes

Among 85 patients with HF related to RHD and CHD, all presented a theoretical indication for cardiac surgery, and 74 were deemed fit for surgery. Intervention was scheduled in 38 patients with RHD (86%) [median 19 years (IQR 12–31)] and in 36 patients (88 %) with CHD [median 4 years (IQR 1–5)]. Eleven patients (13%) presented with co-morbidities or at an advanced stage and were not considered surgical candidates. Data concerning major outcomes and surgical follow up are depicted in Fig. 1.

Twenty-seven patients (14 with RHD and 13 with CHD) were operated on during the study period, accounting for 36% (27/74) of patients deemed suitable for surgery. Selection was based on expected benefit from the surgery, with the most favourable risk:benefit ratio, familial support and patients’ consent. Despite the low surgical risk, two patients suffering from Down syndrome were not considered suitable for heart surgery due to the expected survival rate in deprived areas. Surgery was performed after a median waiting time of 10 months (IQR 6–21) in foreign hospitals funded by NGOs.

Among 14 patients with RHD, 13 (93%) underwent mitral surgery, both replacement (*n* = 10, 77%) and repair surgery (*n* = 3, 23%) such as annuloplasty, implantation of artificial cordae and commissurotomy. Combined mitral and aortic valve replacement was performed in two patients (14%). Tricuspid repair surgery was performed in four patients (28%).

Among 13 patients with CHD, surgical treatment included four cases (31%) of VSD closure, two (15%) of AVSD repair, two (15%) of PDA ligation and one case (8%) of ASD. closure. Four patients (31%) underwent surgery for complex congenital defects (two with tetralogy of Fallot, one with aortopulomonary window, one with persistent truncus arteriosus).

Among 85 patients with HF related to RHD and CHD, 16 (19%) had died by follow up after a median of 38 months (IQR 5–52), and 19 (22%) were lost to follow up (Fig. 1). There were 13 deaths (15%) among patients who did not undergo surgery despite the presence of clear indications, one peri-operative death and two late post-operative deaths due to complications related to mechanical valves (one endocarditis, and one severe brain haemorrhage).

Nineteen patients (22%) were lost to follow up and were considered as not operated, given the lack of access to cardiac surgery in Uganda. Twelve out of the 19 patients lost to follow up were likely to have died due to to advanced disease at the time of diagnosis. All patients who had undergone cardiac surgery experienced improvement in clinical symptoms (22 patients reverting to NYHA class I; two patients reverting to NYHA class II).

## Discussion

We report here the first prospective hospital-based series of HF patients in Uganda and show that RHD, a preventable disease, remains the major cause of HF in a young population. However, CHD was the leading cause among children, especially those under 10 years. Other causes were also identified as hypertensive cardiomyopathy, endomyocardial fibrosis and presumed ischaemic heart disease. A small fraction of young surgical candidates had access to treatment through the efforts of NGOs. We believe our study will add to the knowledge of cardiovascular disease in sub-Saharan Africa, as robust echo-based data are limited,^5^,^15^-^17^ and surgical outcomes seldom depicted.

Regarding the aetiology of HF, our findings are consistent with those of other sub-Saharan countries, as illustrated in Table 3.^4^,^5^,^7^,^15^-^17^ In Uganda, RHD remains the leading cause of HF in young adults and the second cause in children. As in other series, we found that RHD affected mostly young women in the third decade of life, and that mitral regurgitation was the most common presentation.^18^ Diagnosis was often made when left ventricular function was already impaired, requiring intervention.

**Table 3:** Echo-based diagnosis of heart failure in sub-Saharan Africa.

	*Uganda*	*Nigeria^16^*	*South Africa^5^*	*Ghana^4^*	Malawi^17^*	*Cameroon^15^*	*THESUS-HF 7^§^*
Inclusion period	2009–2013	2002–2006	2006	1992–1995	2001–2005	2002–2008	2007–2010
Settings	Kampala	Abuja	Soweto	Accra	Mzuzu	Kumbo	9 nations
Total sample size	272	–	1960			8121	
Population with CVD	190	–			3908*		
Sample size with HF	140	340	844 (de novo)	572	–	462	1006
Age	40 (14–66)	51 ± 15	55 ± 16	42 ± 1	40 ± 32	43 ± 18	52 ± 18
Females, *n* (%)	59	49	57	45	59	43	51
Causes of HF							
First aetiology	RHD	HCMP	HCMP	HCMP	RHD	RHD	HCMP
Second aetiology	CHD	DCMP	DCMP	RHD	HCMP	DCMP	DCMP
Third aetiology	HCMP	RHD	Right HF	DCMP	DCMP	HCMP	RHD

CVD = cardiovascular disease, HF = heart failure, RHD = rheumatic heart disease, HCMP = hypertensive cardiomyopathy, DCMP = dilated cardiomyopathy. *Data from these studies are presented as mean ± SD or median (IQR) as available. In the study by EZ Soliman, the registry did not specifically address the causes of HF, but the main causes of cardiovascular disease. ^§^THESUS-HF = THE SUb-Saharan Africa survey of Heart Failure was a prospective survey of patients with acute HF admitted to 12 university hospitals in nine sub-Saharan countries: South Africa, Mozambique, Uganda (*n* = 154), Kenya, Ethiopia, Sudan, Senegal, Nigeria and Cameroon.

Our study underscores the need for preventive strategies in order to lessen the burden of RHD in Africa. RHD is a preventable disease provided patients receive penicillin for Group A streptococcal pharyngitis (primary prevention) or after a first attack of acute rheumatic fever (secondary prevention). Notwithstanding, RHD remains a major burden in low-income countries, affecting 15 million people and leading to at least 200 000 deaths per annum worldwide.^19^,^20^

Diagnosis is often made when costly interventions are required, leaving most sub-Saharan African patients to the natural course of the disease. Comprehensive programmes focusing on secondary prophylaxis are cost-efficient and could avoid progression to irreversible valve damage. Our study advocates launching preventive strategies in Uganda. The role of echocardiography-based screening in endemic areas is still a matter of controversy.^21^-^24^

In our study, CHD was the main cause of HF among children and accounted for up to 9% of HF among adults, suggesting the need for diagnostic expertise in echocardiography and for cardiac surgery facilities.^9^ While in developed countries prenatal diagnosis is currently used to detect CHD, access to diagnosis and treatment are limited in low-income countries.^25^ Furthermore, simple CHD may be cured by timely surgical or percutaneous treatment, whereas a delayed diagnosis increases morbidity and mortality rates. Efforts should focus on early detection of CHD and on building a referral system for diagnosis, management and follow up of patients in a resource-deprived setting.^25^

In contrast with previous reports,^3^,^26^ endomyocardial fibrosis in the urban area of Kampala no longer seems to be the main cause of HF. That could be ascribed to improved socioeconomic conditions. It must be stressed that the nationwide prevalence of the disease could be higher, as suggested by an echocardiography-based screening study performed in rural Mozambique.^27^ Urban Uganda seems to follow the trend of the epidemiological transition witnessed in many African countries, with the emergence of hypertensive cardiomyopathy and IHD as major causes of HF in adults.^7^,^4^,^16^

The progression to hypertensive cardiomyopathy could be halted through early diagnosis and appropriate treatment, including the reduction of salt intake. Raising awareness among the general population and health workers should therefore become a priority in African countries,^28^ and could be achieved by training primary healthcare practitioners to use simple algorithms to score cardiovascular risk and initiate treatment when needed.

We found that right ventricular failure due to PH was relatively common in adults, in agreement with the results from other African studies.^4^,^5^,^7^,^16^-^18^ Unlike a recent multinational survey including nine countries, we did not diagnose patients with posttuberculosis or HIV-related cardiomyopathy, and we found only one case of post-partum cardiomyopathy.^7^ We encountered no cases of cor pulmonale due to post-tuberculosis lung damage, tuberculosis-related pericarditis or HIV-related cardiomyopathy in spite of HIV being endemic in Uganda (http://www.unaids.org/en/dataanalysis/knowyourepidemic/epidemiologicalfactsheets/).

Although the epidemiology of cardiovascular diseases is usually treated in African countries as a whole,^8^ differences in climate, diet and income may explain apparent discrepancies between regions. The fact that our study was conducted in an urban area may also account for these conflicting results.

As outlined by our series in which only 36% of surgical candidates had access to treatment and 18% died on the waiting list, there is an urgent need for comprehensive service frameworks to improve level of care, and services by NGOs are insufficient to treat all patients in need of treatment. With the exception of South Africa, access to cardiac expertise and heart surgery remains extremely limited in most sub-Saharan countries.^9^ The absence of trained physicians is a barrier to tackling the burden of cardiovascular disease, a growing public health issue in Africa.^29^

Efforts to overcome these hurdles have resulted in two types of humanitarian projects: the creation of well-equipped, on-site healthcare structures, and the transfer of patients, mainly children, with complex diseases to receive highly specialised care abroad. Our programme was based on providing surgery in European centres. However, prospective studies with long-term follow up to clearly define whether these strategies are effective in reducing infant and young adult mortality in the sub-Saharan socio-economic background are warranted.

## Strengths and limitations

The study was conducted by skilled cardiologists who are experienced in the assessment of valvular heart disease, in collaboration with local medical staff who were previously involved in other studies on the causes of HF in Uganda.^3^,^12^ The diagnosis was therefore robust. The prospective nature of the study has enabled us to form a cohort of patients with structural heart disease and to organise follow up. We provide here original and scarce data on the selection and outcomes of heart surgery candidates in a poorly resourced country.

Our results appear to be in agreement with previous data from other sub-Saharan hospital-based registries, with the exception of tuberculosis and HIV-related heart disorders. Our study was based on a prospectively collected patient cohort from a tertiary teaching hospital, which does not allow us to draw conclusions on the prevalence of HF and its associated echocardiographic patterns in the general population. IHD diagnosis was only presumptive because at the time of the study coronary angiography was not available in Uganda.

Finally, the sample size was relatively modest due to the limited time period of the NGO missions and we acknowledge high rates of loss to follow up. Also, we decided not to analyse outcomes according to treatment in RHD and CHD patients, due to methodological constraints (high rate of lost to follow up in a country with no nationwide mortality register, survivor treatment selection bias). Although descriptive, our study complies with the STROBE guidelines.^30^ We attempted to contact every patient, however, remoteness, frequent changes of mobile phones, and cultural boundaries may explain the difficulties in contacting all patients or their next of kin.

## Conclusions

Rheumatic heart disease prevails as the leading cause of heart failure in urban Uganda, and CHD represents an increasing challenge for African practitioners, whereas hypertensive and ischaemic heart disease emerge among elderly adults. Only a minority of young surgical candidates with RHD and CHD have access to treatment. Mortality rate remains high. Cost-effective preventive strategies for RHD and hypertension, rational referral services for early diagnosis, and north–south transfer of skills may lessen the growing burden of cardiovascular diseases in Africa.
